# Active surveillance should be considered for select men with Grade Group 2 prostate cancer

**DOI:** 10.1186/s12894-023-01314-6

**Published:** 2023-09-30

**Authors:** Kelly R. Pekala, Oskar Bergengren, James A. Eastham, Sigrid V. Carlsson

**Affiliations:** 1 Department of Surgery (Urology Service), Memorial Sloan Kettering Cancer Center, 1133 York Avenue, New York, NY 10065 USA; 2 Department of Urology, Uppsala University, Uppsala, Sweden; 3 Department of Epidemiology and Biostatistics, Memorial Sloan Kettering Cancer Center, New York, NY USA; 4 Department of Urology, Institute of Clinical Sciences, Sahlgrenska Academy at University of Gothenburg, Gothenburg, Sweden

**Keywords:** Prostate cancer, Active surveillance, Grade group 2

## Abstract

**Background:**

Treatment decisions for localized prostate cancer must balance patient preferences, oncologic risk, and preservation of sexual, urinary and bowel function. While Active Surveillance (AS) is the recommended option for men with Grade Group 1 (Gleason Score 3 + 3 = 6) prostate cancer without other intermediate-risk features, men with Grade Group 2 (Gleason Score 3 + 4 = 7) are typically recommended active treatment. For select patients, AS can be a possible initial management strategy for men with Grade Group 2. Herein, we review current urology guidelines and the urologic literature regarding recommendations and evidence for AS for this patient group.

**Main body:**

AS benefits men with prostate cancer by maintaining their current quality of life and avoiding treatment side effects. AS protocols with close follow up always allow for an option to change course and pursue curative treatment. All the major guideline organizations now include Grade Group 2 disease with slightly differing definitions of eligibility based on risk using prostate-specific antigen (PSA) level, Gleason score, clinical stage, and other factors. Selected men with Grade Group 2 on AS have similar rates of deferred treatment and metastasis to men with Grade Group 1 on AS. There is a growing body of evidence from randomized controlled trials, large observational (prospective and retrospective) cohorts that confirm the oncologic safety of AS for these men. While some men will inevitably conclude AS at some point due to clinical reclassification with biopsy or imaging, some men may be able to stay on AS until transition to watchful waiting (WW). Magnetic resonance imaging is an important tool to confirm AS eligibility, to monitor progression and guide prostate biopsy.

**Conclusion:**

AS is a viable initial management option for well-informed and select men with Grade Group 2 prostate cancer, low volume of pattern 4, and no other adverse clinicopathologic findings following a well-defined monitoring protocol. In the modern era of AS, urologists have tools at their disposal to better stage patients at initial diagnosis, risk stratify patients, and gain information on the biologic potential of a patient’s prostate cancer.

## Background

As PSA testing has increased the frequency of diagnosing men with low risk prostate cancer [1–5], active surveillance (AS) emerged as a strategy to manage men with low risk disease and avoid overtreatment. AS allows men to postpone or avoid active treatment of prostate cancer to preserve urinary, sexual, and bowel function for many years, if not until the transition to watchful waiting (WW). [6] Several large AS series have documented its oncologic safety and the ten-year prostate cancer specific survival of 96–100% for men with low risk or intermediate risk disease. [7–12] AS is well accepted and the preferred initial management strategy for men with Grade Group 1 (Gleason 3 + 3 = 6) prostate cancer without other intermediate-risk features by many different guidelines organizations including the American Urological Association (AUA), National Comprehensive Cancer Network (NCCN), European Association of Urology (EAU), and National Institute for Health and Care Excellence (NICE). [13–20] AS protocols differ by institution but incorporate serial prostate-specific antigen (PSA) testing, digital rectal examination (DRE), prostate magnetic resonance imaging (MRI) and repeat prostate biopsy to actively monitor the cancer, with plans to switch to curative treatment if there are signs of disease progression or upstaging. In contrast to AS, WW adopts a management strategy of observation with planned administration of non-curative treatment to slow the disease if the patient develops symptoms and evidence of metastatic prostate cancer.

While AS is the management strategy for Grade Group 1, there has not been widespread acceptance of AS for Grade Group 2 (Gleason 3 + 4 = 7) prostate cancer, although these patients were included in many of the original studies. The Grade Group 2 natural history more closely aligns with Grade 1 than Grade 3 prostate cancer when evaluating recurrence free progression following radical treatment with either radical prostatectomy (RP) (with prostate biopsy and RP specimen), Radiation Therapy (RT) +/- androgen deprivation therapy (ADT), or RT alone. [21] A recent study using the US National Cancer Database reported that AS as a treatment option increased from 2.1% in 2010 to 6.8% in 2016 for men with favorable intermediate risk prostate cancer. [22] With accumulative evidence confirming oncological safety, and more sophisticated imaging technology, are select men with favorable intermediate risk, localized prostate cancer suitable candidates for AS? Broadening AS criteria could allow more men to avoid overtreatment and adverse side effects of treatment. Our objective was to review the evidence for AS for Grade Group 2 (Gleason 3 + 4 = 7) prostate cancer as this approach has already been recommended as a management option by many of the guideline organizations. [19, 23–26].

## Methods

We conducted a literature search searching the databases PubMed, Scopus and Embase. The search used the terms: “prostate cancer”, “active surveillance” and “intermediate risk”. The authors retrieved relevant articles in full text. Up-to-date, current urology guidelines were retrieved and reviewed in full text.

## Discussion

Herein, we examined which intermediate risk men with prostate cancer are candidates for AS, the risks and benefits of AS for grade group 2 disease. We also examined how risks can be mitigated and what the best existing evidence demonstrates about AS and oncologic outcomes for this population.

### Current guideline recommendations

The guidelines groups all allow for inclusion of Grade Group 2 disease with slightly differing definitions of risk, largely based on the D’Amico criteria. [27] The AUA/ASTRO/SUO guidelines include men with a low PSA density, low tumor volume, low percentage of Gleason 4 pattern on biopsy, [23] whereas CCO/ASCO includes patients with low volume disease. [24] The NCCN recommends confirmatory prostate biopsy with or without MRI and with or without molecular tumor analysis to establish candidacy. [19] The EAU includes men with < 10% pattern 4, PSA < 10 ng/mL, <cT2a with low volume disease on imaging and biopsy, [25] and NICE more broadly includes men who choose not to have immediate treatment. [26] All guidelines emphasize that candidates for AS with Grade Group 2 should also exhibit other features of favorable intermediate risk including low-volume disease, i.e., a small number, or percentage, of biopsy cores positive, a low PSA < 10 ng/mL and clinical T stage less than T2b. For men with Grade group 2 and cribriform pattern as the grade 4 component, 2022 EAU guidelines do not recommend AS, [28] as this finding is typically associated with adverse pathologic and clinical outcomes. [29] However, as no prospective data exists of Grade group 2 AS cohort(s) with cribiform pattern in the literature and multiple studies restrict the adverse events of cribiform pattern to patients with large cribiform pattern when controlling for other readily available clinical/pathologic parameters, [30, 31] the decision on candidacy for AS in such patients should be made by multidisciplinary consensus.

### Quality of life benefits and discontinuation rates

AS has several obvious benefits for the quality of life in men with prostate cancer—maintaining their current quality of life and avoiding possible treatment side effects such as erectile dysfunction, urinary incontinence from surgery or radiation for as long as possible. AS protocols with close follow up always allow for an option to change course and pursue curative treatment. Some may argue that the time men with Grade Group 2 disease spend on AS is not long enough to gain the quality of life benefits. However, a recent systematic review of prospective and retrospective AS cohorts found that there was no difference in rates of deferred treatment between men with low-risk prostate cancer on AS and men with intermediate risk Grade Group ≤ 2. The 10-year treatment-free survival was between 19 and 69% for intermediate risk patients (inclusive of Grade Group 2 and 3). [32] This suggests that many men may be able to stay on AS until the transition to watchful waiting. Treatment-free survival in the MSK AS cohort of men with Grade group 2 disease was 61% (95% CI 52–70%) at 5 years and 49% (95% CI 37–60%) at 10 years. [33] This suggests that most men who will progress or have pathologic upstaging are found within the first few years of AS with strict protocols and that men who do not progress can potentially benefit for many years.

### Oncologic outcomes

Much of the resistance to incorporating AS for men with Grade Group 2 disease may be based on concern for oncologic outcomes. In terms of level 1 evidence, there are three randomized trials that included men with intermediate risk prostate cancer, in two of them, men were managed by WW [34, 35] and in one, men were actively monitored with PSA alone. [36] In the two trials that compared WW to RP (SPCG-4 and PIVOT), for men with intermediate risk disease, with active treatment there were reductions in prostate cancer metastasis by 20%, prostate cancer mortality by 24%, and overall mortality by 16% in the SPCG-4 trial and a 10% reduction in in overall mortality in the PIVOT trial. As these trials are older, they notably did not distinguish between unfavorable and favorable intermediate risk disease. None of these trials were able to incorporate modern multiparametric prostate MRI, individual patient risk stratification tools such as the UCSF-CAPRA or MSKCC prostate cancer nomograms, or new molecular tumor analyses to provide improved staging accuracy and better information of the biologic potential of their prostate cancer. The SPCG-4 trial was conducted in the pre-PSA era, so most men had palpable disease. [34] In the more recent ProtecT trial that compared RP to Radiation Therapy (RT) and active monitoring with PSA levels, there were approximately 100 men with intermediate risk disease in each cohort. At 10 years of follow up and 15 years of follow up, there were no significant differences in the number of deaths from prostate cancer by treatment arm and disease risk at diagnosis. [36, 37] Patients in the active monitoring group had a higher cumulative incidence of disease clinical stage progression and histologic upstaging compared to the RP and RT cohorts [37, 38] and higher rates of metastasis than in the surgery or RT group. [36, 37] However, the active monitoring group was monitored with PSA levels every 3 months for the first year and then a PSA every 6 months and then at the frequency of investigator’s discretion. There was no repeat biopsy, so this active monitoring group is not equivalent to modern AS today. [39].

In more recent observational cohorts, with median follow ups of 1.8–8.2 years, the risk of prostate cancer specific mortality ranged from 0 to 10% for intermediate risk patients with a variety of inclusion criteria. [11, 12, 40–46] Notably, the more recent observational cohorts that incorporate the newer technologies into their AS protocol report lower rates of prostate cancer deaths with 3% in a Canadian cohort [47] and 0% in the MSKCC series. [48] In terms of metastasis-free survival, the more recent observational studies suggest rates of 0-3.6% for men with intermediate risk disease depending on length of follow up. [11, 33, 40–42, 44, 46, 49]. An older study that was prior to the MRI era, with a less stringent repeat biopsy protocol at every 3–4 years had higher rates of metastasis of 16% for men with intermediate risk disease. [44] So while critics of AS for Grade Group 2 disease may point to higher rates of metastasis and prostate cancer specific mortality in historical cohorts from randomized controlled trials, the landscape of prostate cancer care is rapidly changing. The older trials such as SPCG-4, PIVOT did not distinguish between favorable and unfavorable intermediate risk, when they identified a benefit to RP over WW. [34, 50] Even in the ProtecT trial, the active monitoring regimen was based on PSA triggers rather than a pre-specified protocol and there were higher rates of progression and metastasis in this group. [10] A recent systematic review and meta-analysis that included prospective and retrospective studies compared AS outcomes for men with low risk versus intermediate risk prostate cancer with Grade Group ≤2 and found no significant differences in rates of metastasis, RR 2.09, (95% CI 0.75–5.82) and no prostate-cancer related deaths in the cohorts. [32]

As illustrated in several large-scale randomized trials as well as prospective and retrospective studies, men with very low and low risk prostate cancer, Grade Group 1 (Gleason 3 + 3 = 6) without other intermediate risk features, are certainly AS candidates, since long-term risks of metastasis or death from prostate cancer are rare. On the other hand, currently men with unfavorable intermediate risk—high-volume Grade Group 2 or Grade Group 3 prostate cancer should *not* be recommended AS as their risks of poor oncologic outcomes when managed expectantly are considerably higher over time. The recent report from the ProtecT trial found no statistically significant differences in prostate cancer specific mortality at 15 years of follow-up between men with localized prostate cancer treated with prostatectomy, radiation or active monitoring, however, the risk of metastasis was double in the active monitoring group. [37] A recent systematic review and meta-analysis that compared low risk to intermediate risk (inclusive of Grade Group 3) found an increased risk of metastasis, RR 5.79 (95% CI 4.61–7.29) and prostate cancer-related death, RR 3.93 (95% CI 2.93–5.27) which is consistent with the prior randomized controlled trials. However, when they restricted their analysis to low risk versus intermediate risk (Grade Group 2), they found no statistically significant difference in rates of metastasis and no prostate cancer specific deaths. [32] Men with favorable intermediate risk prostate cancer who have low-volume Grade Group 2 fall in between low and unfavorable intermediate risk on the risk spectrum. Thus, they can benefit and should be considered for AS as a viable management option but should be carefully staged and monitored. Also of vital importance is appropriate patient counseling on the risks of AS, particularly that of metastasis and progression, as the ideal AS protocol has not been established.

### Pathologic upstaging

Critics may also point to the large proportion of men that will ultimately go onto radical treatment of their disease or have pathologic upstaging while on AS and argue that perhaps this time on AS is not “worth it.” They may also point to the high rates of termination of AS within 2 years. However, the pathologic upstaging based on biopsy reclassification, PSA, MRI can be expected and indicates that the AS protocol is appropriately identifying patients whose prostate cancer warrants treatment and would benefit from treatment such as the Grade Group 3. In addition, the rates of deferred treatment for Grade Group 2 are similar to that of Grade Group 1. [32] Providers should use shared decision making and incorporate the patient’s preferences when assisting with decision-making to find an option that balances oncologic control and quality of life.

### Future directions for AS

In the modern era, urologists have many more tools at their disposal to assist with the initial staging of localized prostate cancer and inform on the biologic potential of a patient’s personal disease. As the guidelines criteria suggest, clinical stage, PSA levels, % biopsy cores positive, presence of cribiform or intraductal pathologic features all currently factor into the AS eligibility criteria. Other aspects that should be considered include family history, germline mutations, MRI findings, and quantification of Gleason pattern 4. Most current AS protocols include a confirmatory biopsy and use of prostate MRI to accurately stage the disease. In addition to the existing eligibility criteria, future active surveillance protocols may incorporate additional information such as quantification of pattern 4 and molecular biomarkers. The amount (millimeters) of pattern 4 is associated with risks of adverse pathology and biochemical recurrence after RP. [51] In addition, molecular biomarkers molecular (e.g., Decipher, Oncotype Dx, Prolaris or Promark) may improve risk stratification and treatment decision making in some cases. [52, 53].

## Conclusions

Taken together, the available evidence suggests that AS is a viable option for carefully selected and well-informed patients with favorable intermediate risk Grade Group 2 prostate cancer who are monitored closely under an established AS protocol. It provides evidence that select men are suitable for an initial period of close AS and deferred treatment based on the growing evidence of the safety of this approach. This conclusion is drawn based on observations of outcomes in relation to disease aggressiveness, primarily the architectural pattern of prostate cancer under the microscope, i.e., Grade Groups (or Gleason Score), which remains the best predictor of cancer-specific outcomes in men with prostate cancer. [21] More prospective studies are needed to better evaluate Gleason pattern 4 quantification, liquid biopsy and molecular tumor analysis and its utility for AS, for this cohort.

## Data Availability

N/A.
